# Editorial: Computational approaches for interpreting experimental data and understanding protein structure, dynamics and function relationships

**DOI:** 10.3389/fmolb.2022.1018149

**Published:** 2022-10-03

**Authors:** Kaifeng Hu, Woonghee Lee, Gaetano T. Montelione, Nikolaos G. Sgourakis, Beat Vögeli

**Affiliations:** ^1^ Innovative Institute of Chinese Medicine and Pharmacy, Chengdu University of Traditional Chinese Medicine, Chengdu, Sichuan, China; ^2^ Department of Chemistry, University of Colorado Denver, Denver, CO, United States; ^3^ Department of Chemistry and Chemical Biology, Center for Biotechnology and Interdisciplinary Sciences, Rensselaer Polytechnic Institute, Troy, NY, United States; ^4^ Department of Pathology and Laboratory Medicine, The Children’s Hospital of Philadelphia, and Department of Biochemistry and Biophysics, Perelman School of Medicine, University of Pennsylvania, Philadelphia, PA, United States; ^5^ Department of Biochemistry and Molecular Genetics, University of Colorado Anschutz Medical Campus, Aurora, CO, United States

**Keywords:** biomolecules, data acquisition and analysis, experiment-aided computation, algorithms, multi-scale simulation and application, software and web services, database, structural biology

The three-dimensional (3D) structure and dynamics of a biomolecule are keys to understanding its function. A variety of experimental structural biology techniques capable of determining biomolecular 3D structures and dynamics at atomic resolution have been developed, including X-ray crystallography, NMR, and cryo electron microscopy (cryoEM). Using these methods, atomic coordinate sets for more than 180,000 biomolecules have been determined and archived in the worldwide Protein Data Bank (wwPDB). Sequences for billions of proteins are also available in genomic sequence databases. However, these data are only the starting point for structure-function studies aimed at testing specific hypotheses and understanding mechanisms underlying biological processes. The exponential growth of computing power and algorithms now enables multiple computational approaches for interpretation of these data, and for simulation of biological processes.

In this Research Topic, entitled “*Computational Approaches for Interpreting Experimental Data and Understanding Protein Structure, Dynamics and Function Relationships*,” we have aimed to cover promising, recent, and novel research and technology development interfacing experimental and computational methods directed to structural, dynamic, and functional studies of biomolecules. Six different articles have been contributed from our colleagues, and one of them is from the group of Prof. Montelione, who also served as one of guest editors for this Research Topic.

Transthyretin (TTR) amyloidosis is known to cause different human diseases including senile systemic amyloidosis and familial amyloid cardiomyopathy/polyneuropathy. Prof. Jin Hae Kim, Prof. Wookyung Yu and their group members studied structural ensembles of TTR by machine-learning based nuclear magnetic resonance (NMR) chemical shift prediction and molecular dynamics (MD) simulation in the contributed paper entitled “*Aggregation-Prone Structural Ensembles of Transthyretin Collected With Regression Analysis for NMR Chemical Shift*” (Yang et al.). They suggest the correlation of the structural deformation of the DAGH β-sheet and the AB loop regions to the manifestation of the aggregation-prone conformational states of TTR. This suggestion has been cross-validated by circular dichroism (CD) spectroscopy and NMR order parameter analysis.

Another emerging area of protein structure analysis involves combining advanced modeling methods with sparse experimental data, like that obtained by NMR using perdeuterated samples of biomolecules. These methods were assessed as part of the Critical Assessment of Protein Structure Prediction (CASP) (Kuenze and Meiler, 2019; Robertson et al., 2019; Sala et al., 2019). Among the best performing methods in CASP13 was MELD (modeling employing limited data). MELD uses Bayesian inference to integrate data from different experimental sources with an atomistic force field to predict structures (MacCallum et al., 2015; Perez et al., 2016) and is well suited to handle sparse, highly ambiguous restraints. In “*Simultaneous Assignment and Structure Determination of Proteins From Sparsely Labeled NMR Datasets*” (Mondal and Perez), Mondal and Perez describe the MELD-NMR pipeline, together with improvements that provide more accurate models for several CASP “NMR-guided” targets than observed in the original CASP13 study. MELD-NMR provides a significant improvement over previously described approaches for structure determination with ambiguous, sparse, and noisy NMR data.

Intrinsically disordered proteins, or IDPs, are an important class of biomolecules with key biological functions. It is estimated that approx. 40% of proteins encoded by the human genome contain an IDP segment of at least 30 residues. In this mini-review, Czaplewski et al. outline recent developments in modeling ensembles of this important class of proteins from a diverse set of experimental observables, including sparse NMR data, SAXS, and XL-MS. The use of time- and ensemble-averaged methodologies are discussed, with emphasis on computational approaches for determining both an ensemble of representative conformers, and their dynamics.

Large amounts of NMR data in diverse format are great resources for NMR structural biology studies, but can create a burden for users to explore and examine them. There is an urgent need for tools for rapid access and translation of the large amount of NMR data in diverse formats. Further, federation of different data resources and powerful computational approaches, such as advanced statistical studies and machine learning, can extend the information in the existing data resources and unveil possible latent insights. Profs. Eghbalnia and Hoch and their group members describe the NMRbox which merges NMR data resources and computation power to facilitate data-centered research in the contributed paper entitled “*Merging NMR Data and Computation Facilitates Data-Centered Research*” (Baskaran et al.). The NMRbox can integrate diverse data resource and create a data lake, called ReBoxitory, which can provide facile and local access to time-stamped copies of high-quality data resources from multiple databases for NMR structural biology. In addition, combination of data repository (BMRB, PDB, etc.) with the NMRbox computational platform can speed and simplify computational workflows. The NMRbox platform creates an environment for developing meta-software and supporting complex workflows. It can foster data interoperability, semantic data management, and reproducible research.

In the contributed paper entitled “*Concurrent Identification and Characterization of Protein Structure and Continuous Internal Dynamics with REDCRAFT*” (Omar et al.), Prof. Valafar and his group members developed and benchmarked the concurrent characterization of protein structure and dynamics using the residual dipolar coupling (RDC) analysis software REDCRAFT (Cole et al., 2021). Structures of dihydrofolate reductase (DHFR), a 159-residue protein whose internal dynamics have been described by a mixed mode model of internal dynamics, were calculated by three different methods: using traditional Ramachandran restraint, using context-specific dihedral restraints generated by PDBMine, and using the Dynamic Profile generated by REDCRAFT. The Dynamic Profile provided identification of different dynamical regions of the protein. The utilization of the Dynamic Profile outperformed the other two methods by identifying the dynamic regions and assembling relatively rigid fragments.

In the contributed paper “*AlphaFold Models of Small Proteins Rival the Accuracy of Solution NMR Structures*” by Tejero et al., AlphaFold models of six small proteins, together with the corresponding experimental NMR and X-ray crystal models, were assessed against experimental NMR data (Tejero et al.). While Prof. Montelione is on the editorial board for this Research Topic, Prof. Francesca Marassi from Sanford Burnham Prebys edited this article. The model validation analysis used multiple server-based structure validation tools, including Protein Structure Validation Software suite (PSVS) (Bhattacharya et al., 2007) integrating several knowledge-based structure validation tools, as well as model vs. data validation using NOESY peak lists (RPF-DP scores) (Huang et al., 2005), protein rigidity and chemical shift (ANSURRS scores) (Fowler et al., 2020), and ^15^N-^1^H residual dipolar coupling data (RDC Q factors) (Cornilescu et al., 1998). AlphaFold models were observed to fit to the NMR data as well as, or in some cases better than, “experimental models” generated from these same data and previously deposited in the Protein Data Bank. Additionally, the AlphaFold models of two target proteins from the Critical Assessment of Protein Structure Prediction (CASP) (Huang et al., 2021), which were not used in the original training of AlphaFold, were also observed to fit remarkably well to the experimental NMR data. However, the AlphaFold model of a third CASP target, which exhibits significant conformational dynamics in solution, was not as good a fit to experimental data. The authors suggest that AlphaFold can accurately model small, relatively rigid protein structures in solution, and can often be used reliably for guiding experimental NMR data analysis.

Overall, different groups have contributed articles with different perspectives aimed at the same goal—viz, our knowledge-limits of important biological processes can be greatly expanded, and the invisible world explored, by interpreting experimental data using advanced computational methods. To do so, it is important to establish reproducible, easy-to-use integrated research environments, which can accelerate sustained and progressive scientific advances. By making advanced, reliable computational tools more accessible to the broad scientific community, we hope to enable novel, and in some cases unanticipated, scientific discovery.
